# A Study and Review of Effects of Botulinum Toxins on Mast Cell Dependent and Independent Pruritus

**DOI:** 10.3390/toxins10040134

**Published:** 2018-03-23

**Authors:** Roshni Ramachandran, Marc J. Marino, Snighdha Paul, Zhenping Wang, Nicholas L. Mascarenhas, Sabine Pellett, Eric A. Johnson, Anna DiNardo, Tony L. Yaksh

**Affiliations:** 1 Department of Anesthesiology, University of California, San Diego, La Jolla, CA 92093, USA; roramachandran@ucsd.edu (R.R.); mjmarino@ad.ucsd.edu (M.J.M.); spaul@westernu.edu (S.P.); 2 Department of Medicine, Division of Dermatology, University of California, San Diego, La Jolla, CA 92093, USA; zhenping.w@gmail.com (Z.W.); nmascare@ucsd.edu (N.L.M.); adinardo@ucsd.edu (A.D.); 3 Department of Bacteriology, University of Wisconsin, Madison, WI 53706, USA; sabine.pellett@wisc.edu (S.P.); eric.johnson@wisc.edu (E.A.J.)

**Keywords:** botulinum toxin, itch, SNARE, VAMP, mast cells, compound 48/80, chloroquine

## Abstract

Pruriceptive itch originates following activation of peripheral sensory nerve terminals when pruritogens come in contact with the skin. The ability of botulinum neurotoxins (BoNTs) to attenuate transmitter release from afferent terminals provides a rationale for studying its effect on pruritus. This study investigated the effects of BoNT/A1 and BoNT/B1 on mast cell dependent (Compound 48/80:48/80) and independent (Chloroquine:CQ) scratching. C57Bl/6 male mice received intradermal injection of 1.5 U of BoNT/A1, BoNT/B1 or saline 2, 7, 14 and 21 days prior to ipsilateral 48/80 or CQ at the nape of the neck. Ipsilateral hind paw scratching was determined using an automated recording device. The effect of BoNTs on 48/80 mediated mast cell degranulation was analyzed in human and murine mast cells and the presence of SNAREs was determined using qPCR, immunostaining and Western blot. Pre-treatment with BoNT/A1 and BoNT/B1 reduced 48/80 and CQ induced scratching behavior starting on day 2 with reversal by day 21. Both serotypes inhibited 48/80 induced mast cell degranulation. qPCR and immunostaining detected SNAP-25 mRNA and protein, respectively, in mast cells, however, Western blots did not. This study demonstrates the long-lasting anti-pruritic effects of two BoNT serotypes, in a murine pruritus model using two different mechanistically driven pruritogens. These data also indicate that BoNTs may have a direct effect upon mast cell degranulation.

## 1. Introduction

Pruritus or itch is an unpleasant sensation that promotes scratching as a primary response. Chronic itch is a debilitating and dominating symptom accompanying several disorders including skin conditions such as atopic dermatitis (AD) as well as in systemic (renal and liver failure) [1,2,3] and neurological disorders (diabetic neuropathy and shingles) [4,5]. Pruriceptive itch, as seen in AD, originates following the activation of peripheral sensory nerve terminals associated with allergic reactions induced by insect bites or when pruritogens come in contact with the skin. Among the several subtypes of primary afferent nerve fibers, a role for C-fibers has been demonstrated in detecting and transmitting pruriceptive signals to the neuraxis [6,7]. 

Many forms of itch are mediated by histamine released from mast cells that activate a subset of neurons expressing TRPV1 receptors as evidenced by the effects of TRPV1 antagonism in histamine evoked activation of dorsal root ganglion (DRG) neurons [8] and reduced histamine evoked scratching behavior [9]. Pruritogens, such as chloroquine (CQ), induce itch via mast cell-independent pathways [10]. Mas-related G protein coupled receptor (Mrgpr) has emerged as a novel class of receptors in histamine independent itch pathways and MrgprA3 is the receptor for CQ. In contrast to histamine dependent pathways, where TRPV1 functions downstream of histamine receptors to promote itch, the histamine-independent pathway utilizes TRPA1 as a key transduction channel downstream of the MrgprA3 receptor [11,12]. 

Botulinum neurotoxins (BoNTs), including the A1 (Botox) and B1 (Myobloc) serotypes, attenuate neurotransmitter release in neurons by the cleavage of terminal soluble N-ethylmaleimide-sensitive-factor attachment protein receptors (SNAREs) [13,14,15]. Data indicate that when BoNT/A1 and B1 are given subcutaneously in the paw, the toxin is taken up in the peripheral terminal and transported back to the central terminal of the primary afferent [16,17]. Studies from our lab as well as from other groups have shown that both subcutaneous (sc) BoNT/A1 and BoNT/B1 reduce local intradermal capsaicin evoked flares in animal [17,18] and human models [19,20,21,22], reflecting the local inhibitory effect upon release of vasodilatory peptides (substance P (sP)/ calcitonin gene-related peptide (CGRP)) from the peripheral terminal evoked by TRPV1 receptor. In addition, following peripheral delivery of BoNTs, cleaved SNAREs are detected in the dorsal root ganglia and dorsal horn along with an associated block of sP release [17]. While BoNTs primarily seem to affect motor neurons in botulism, it is well known that BoNTs can efficiently enter and block neurotransmission in other neuronal subpopulations as well. However, entry and effects on non-neuronal secreting cells, such as mast cells, are less explored, in part because the vesicle release machinery utilizes different (non-neuronal) SNARE proteins that based on the literature are not the targets of medically employed BoNTs. However, an anti-pruritic effect of BoNT/A1 has been demonstrated clinically in several skin disorders, including dermatitis [23], burn induced itch [24], and lichen simplex [25], a localized variant of AD in which acetylcholine appears to be a dominant pruritic mediator. BoNT/A1 also reduced the itch intensity, blood flow and neurogenic inflammation in response to the histamine prick test in human skin [19]. These results jointly suggest the use of BoNTs in treating pruritus, although the mechanism of action remains unknown including whether observed effects are a result of direct action of the BoNT on mast cells or an indirect effect via neurons. The present study demonstrates anti-pruritic effects of BoNT/A1 and BoNT/B1 on histamine dependent compound 48/80 and histamine-independent CQ-induced scratching behavior in mice, and for the first time shows an effect of the BoNTs on cultured murine and human mast cells.

## 2. Results

### 2.1. BoNT/A1 and BoNT/B1 Injection Reduced 48/80 and CQ Induced Scratching 

**Figure 1:**
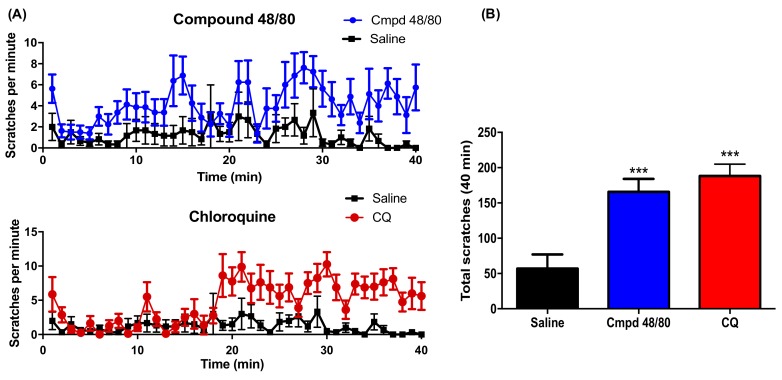
Compound 48/80 and chloroquine induced scratches: (**A**) time course of scratching induced by intradermal injection of compound 48/80 (50 μL of 1 mg/kg) (*N* = 8) or Chloroquine (50 μL of 2mg/mL) (*N* = 8) over a period of 40 min (CQ); (**B**) histogram showing cumulative scratch count following compound 48/80 and CQ in 40 min. All data are expressed as Mean ± SEM. *** *p* < 0.001 as compared to the saline treated group (*N* = 7).

Behavioral responses were recorded for 40 min in the C57Bl/6 mice following intradermal injection of 48/80 and CQ at the nape of the neck. Both pruritogens injected unilaterally induced ipsilateral scratching behavior. The total number of scratches in the 40 min period increased significantly following intradermal injection of mast cell-dependent 48/80 and mast cell-independent CQ (Figure 1).

Bouts of scratching induced by 48/80 and CQ were reduced by 1.5 U of ipsilateral BoNT/A1 and BoNT/B1 given locally (intradermal) two days prior to the intradermal injections of pruritogens. Analysis of total scratching in the 40 min period showed that this reduction was statistically significant. Importantly, the 1.5 U of intradermal BoNT-A1 or BoNT-B1 did not produce detectable alterations in motor function or strength. Animals displayed normal grasping behavior as measured by a suspension test where the animals were required to grip onto the wire mesh for at least 1 min and showed normal hind limb placing and stepping reflexes [17].

### 2.2. BoNT/A1 and BoNT/B1 Have a Long Duration of Effect in Reducing Compound 48/80 and CQ Induced Scratching

**Figure 2:**
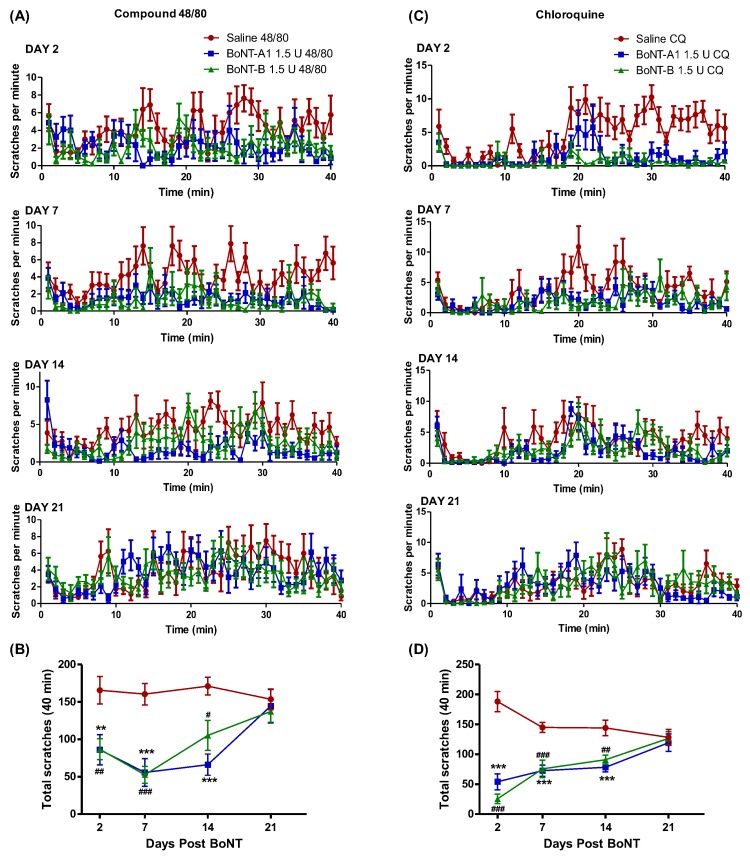
Duration of action of BoNT/A1 and BoNT/B1 in reducing compound 48/80 and chloroquine induced scratching: Mice were treated with intradermal saline (control), BoNT/A1 (1.5 U), or BoNT/B1 (1.5 U) at 2, 7, 14, or 21 days prior to intradermal administration of compound 48/80 (**A**,**B**) or Chloroquine (**C**,**D**). The total scratches per minute were observed over a 40 min time interval after administration of compound 48/80 or chloroquine. Plots indicate mean ± SEM for cumulative flinches observed at days 2, 7, 14 and 21. ** *p* < 0.01, *** *p* < 0.001 vs. saline; ^#^ *p* < 0.05, ^##^ *p* < 0.01, ^###^ *p* < 0.001 as compared to saline, *N* = 8 animals per group.

One of the hallmarks of pharmaceutical BoNTs is their long duration of action, lasting 2–6 months in humans after intramuscular injection. Local intramuscular injection of BoNT/A1 in mice results in local paralysis that peaks at day 2 after injection and slowly decreases in effect over the following 2–3 weeks [26]. To determine whether effects of BoNT/A1 and B1 on 48/80 and CQ induced scratching have a similarly long-lasting duration, 1.5 U BoNT/A1 or BoNT/B1 or saline were given on days 2, 7, 14 and 21 days prior to administration of 48/80 and CQ treatment on the same side of the neck. BoNT/A1 and BoNT/B1 significantly reduced 48/80 induced scratching behavior on days 2, 7 and 14, but not on day 21 as compared to the saline treated group, suggesting a reversal of effect of BoNT by day 21 (Figure 2). A similar long-lasting effect of BoNT/A1 and B1 was observed on CQ induced scratching as well, where pretreatment with unilateral BoNT/A1 and B1 significantly reduced CQ induced scratching behavior on days 2, 7 and 14 with a complete reversal by day 21 (Figure 2). In both cases, a slow recovery to normal scratching behavior was observed over time, similarly as is seen with muscle paralysis after BoNT treatment. Interestingly, even though BoNT/A1 has a significantly longer duration of action than BoNT/B1 in causing muscle paralysis, in the pruritus assay, both toxins had a similar duration of action in suppressing 48/80 or CQ induced scratching behavior.

### 2.3. BoNT/A1 and BoNT/B1 Reduce Compound 48/80 Induced Murine and Human Mast Cell Degranulation

**Figure 3:**
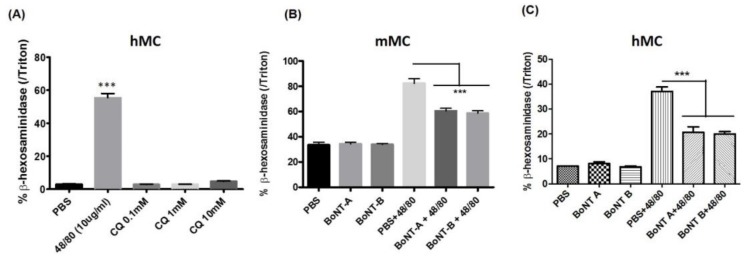
Effect of BoNT on compound 48/80 induced mast cell degranulation in human (hMC) and mouse mast cell culture (mMC): Mast cell β-hexosaminidase release (as index of mast cell degranulation) following compound 48/80 (48/80, 10 μg/mL) and chloroquine (CQ) (**A**); mast cell β-hexosaminidase release induced by 48/80 (1μg/mL) 24 h after treatment with 0.5 U of BoNT/A1 or BoNT/B1 in murine mast cell culture (**B**) and in human mast cell culture (**C**). *** *p* < 0.001 compared to other groups.

The inhibitory effects of BoNT/A1 and B1 on 48/80 induced scratching observed in the in vivo studies could be due to a direct effect of the BoNTs on mast cells or secondary to the inhibition of mediator release from primary afferents inhibited by BoNTs. In order to determine whether BoNT/A1 and B1 directly affected the functioning of mast cells, an in vitro assay using isolated murine and human iPSC derived mast cells was performed. The primary murine mast cells were treated with 48/80 for 20 min at 37 °C, leading to degranulation as evidenced by β-hexosaminidase release compared to untreated control cells. As expected, treatment with CQ did not induce mast cell degranulation. Interestingly, pre-treatment of both murine and human mast cells with 0.5 U of BoNT/A1 and /B1 for 24 h significantly reduced 48/80 mast cell degranulation (Figure 3). This indicates a direct effect of BoNT/A1 and B1 on mast cells.

### 2.4. Western Blot Analysis of Expression and Effect of BoNT/A1 and BoNT/B1 on SNAP-25 and VAMP 1/2/3 in Human and Murine Mast Cells

**Figure 4:**
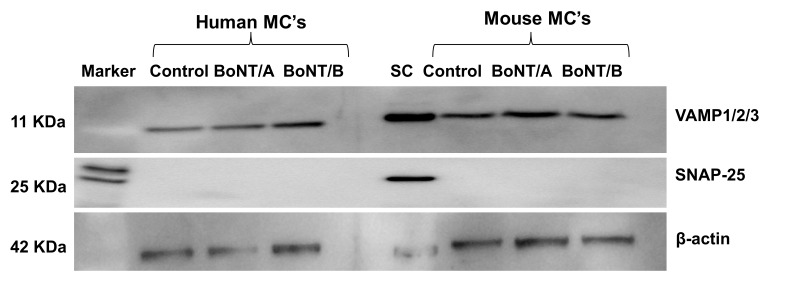
Expression of SNARE proteins in mast cells: Representative image of Western blots showing expression of SNAP-25 or VAMP-1/2/3 in the mast cells with or without BoNT/A1 or B1 treatment (0.5 U for 24 h). Spinal cord (SC) tissue was used as a positive control for SNAP-25 expression. Mast cells did not express SNAP-25 and hence no effect of BoNT/A1 on SNAP-25 was observed. VAMP 1/2/3 were expressed in mast cells; however, they were not affected by pre-treatment with BoNT/B1; this was repeated three times.

The cellular target of BoNT/A1 is the neuronal SNAP-25 and for BoNT/B1 is VAMP-1 and 2, which are essential components in the neuronal vesicle release machinery. The BoNTs cleave their respective target SNARE proteins, which is the mode of action by which BoNTs block neurotransmitter release. While the degranulation machinery identified in mast cells utilizes SNAP-23 and VAMP-7/8, which are not cleaved by BoNT/A1 and /B1, the observed inhibition of degranulation of mast cells by BoNTs indicates a mechanism other than SNARE cleavage. In order to confirm this in our model, cultured murine and human mast cells were treated with BoNT/A1 and BoNT/B1 for 24 h and SNAP-25 and VAMP isoforms were analyzed using Western blot. Consistent with previous reports, Western blot analysis did not detect expression of SNAP-25 (Figure 4). It should be noted that spinal cord samples loaded as a positive control on the same immunoblot membrane clearly showed SNAP-25 expression, suggesting that mast cells do not express SNAP-25 at detectable levels. Though expression of VAMP-1/2/3 was observed in mast cells, BoNT/B1 surprisingly did not reduce full-length VAMP 1/2/3 levels in these BoNT treated mast cells, suggesting no cleavage of BoNT-B1 specific VAMP proteins.

### 2.5. Expression of SNAP-25 and VAMP 1/2/3 Cleavage with or without BoNT/A1 or B1 Treatment, Respectively, in Human and Murine Mast Cells

**Figure 5:**
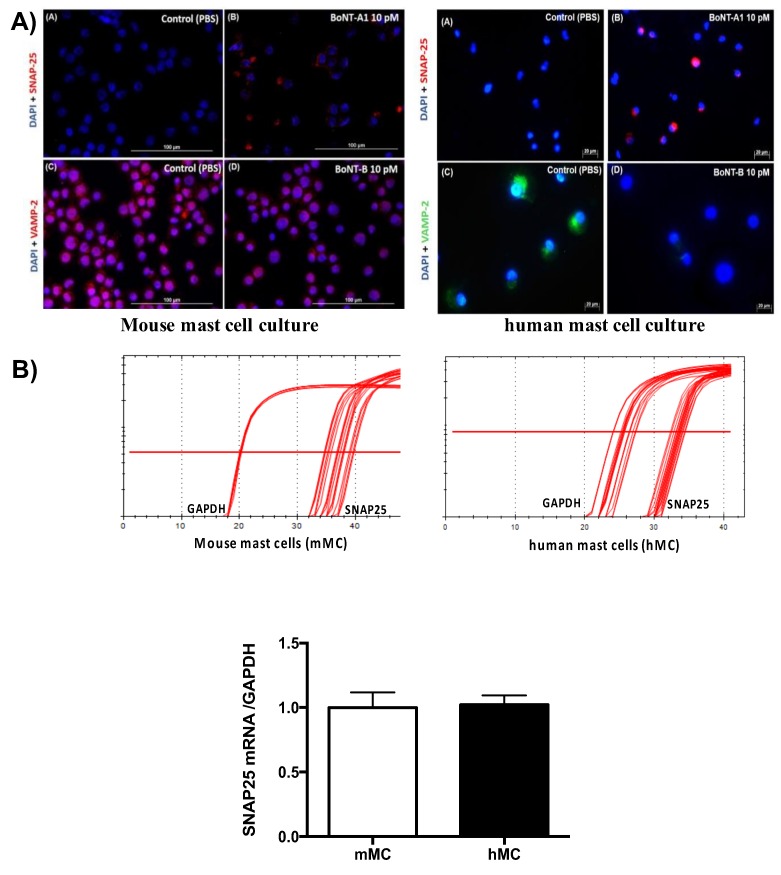
Detection of cSNAP-25 and VAMP-2, respectively, in mouse and human mast cell culture: (**A**) representative images of BoNT/A1-cleaved SNAP-25 and VAMP-2 immunostaining following treatment with BoNT/A1 and BoNT/B1 (10 pM); (**B**) RT-qPCR of SNAP-25 expression in mouse and human mast cell culture. *N* = 3.

Although Western blot did not detect any expression of SNAP-25 in human and mast cell culture, immunostaining was able to detect cleaved products of SNAP-25 following BoNT/A1 treatment on both human and murine mast cells. The SNAP-25 antibody used in the present study detects only the cleaved products (cSNAP-25). The control groups of murine and human MC did not show cSNAP-25 staining; however pre-treatment of the mast cells with BoNT/A1 for 4 h showed a dose-dependent increase in cSNAP-25 staining. DAPI was used to stain the nuclei of the mast cells (Figure 5A). RT-qPCR analysis on human and mouse mast cells showed expression of SNAP-25 mRNA, suggesting the presence of at least low levels of SNAP-25 in mast cells (Figure 5B). The VAMP antibody used recognizes the intact molecule. Therefore, reduction of VAMP protein expression was used as a measure of VAMP cleavage. In control animals, VAMP expression was observed in the control group along with the DAPI stained nuclei. Following pre-treatment with BoNT/B1, the cells showed a reduction in VAMP expression. Thus, VAMP cleavage was significantly greater in the BoNT/B1 treated group as compared to PBS control (Figure 5A). 

## 3. Discussion

The present study demonstrated the anti-pruritic effects of two well characterized and clinically employed BoNT serotypes, Botulinum Toxin A1 (Botox©) and Botulinum Toxin B1 (MyoBloc©) over time in a murine pruritus model using two different mechanistically driven pruritogens. While involvement of SNARE proteins in release of pruritus stimulating mediators from mast cells has been demonstrated [27,28], effects of BoNT/A1 and BoNT/B1 on mast cell release has not been explored. The present study showed that BoNTs may have a direct effect on mast cells in altering its local degranulation, and that this effect may be independent of SNARE cleavage.

### 3.1. Mast Cell Dependent and Independent Pruritogens

Pruriceptive itch is induced peripherally due to the activation of nerve fibers located in the epidermis and is the type of itch observed in several dermatological conditions such as atopic dermatitis, psoriasis, etc. [29]. Pruriceptive itch can be experimentally triggered by several exogenous and endogenous substances. In the present study, we utilized two pruritogens, mast cell dependent, 48/80 and mast cell independent, CQ. Compound 48/80 degranulates mast cells to release histamine [30], which induces itch by binding to and activating C-fibers via gating the TRPV1 channel [8,9]. Other mediators released from mast cells such as serotonin, bradykinin, and prostaglandin can potentiate the effects of histamine induced itch sensation [31]. CQ, on the other hand, elicits itch in a mast cell independent pathway, presumably by activating Mrgpr /TRPA1 receptors [11,12]. A recent study shows that 48/80 may have a direct action on neurons in addition to degranulating mast cells [32] and the interpretation of the results in this study does consider this possibility. However, it should be noted that a previous study has reported mast cell mediated neuronal activity of 48/80 [33]. 

### 3.2. Anti-Pruritic Effect of Botulinum Toxin

BoNT/A1 and BoNT/B1 have been extensively used in clinical and pre-clinical studies for elucidating the mechanisms by which they can inhibit pain transduction in several pain related disorders. Ample evidence now suggests that BoNTs influence release of several neurotransmitters such as acetylcholine, glutamate, CGRP, sP, and serotonin [34,35,36]. The release of these neurotransmitters may play a contributing role in induced itch [37,38]. Patients with AD show an increase in density of nerve fibers containing CGRP and sP [39]. Furthermore, pre-clinical studies in pain models have shown that peripheral BoNT can block the release of neurotransmitters from the local afferents as well as from the central nerve terminals [17,18,40,41,42,43], suggesting a possible pathway in which BoNTs may influence the transmission of itch signals to higher brain centers. Our data from the present study suggests that both BoNT/A1 and BoNT/B1 significantly inhibited the scratching behavior induced by two mechanistically different pruritogens. These findings are in accordance with a clinical study showing that BoNT/A1 could reduce the histamine pin prick induced itch intensity in human skin along with diminished blood flow and neurogenic inflammation [19]. Further clinical studies have suggested anti-pruritic effects of BoNT/A1 on conditions accompanied with itch such as lichen simplex, rhinitis, inverse psoriasis, burn induced itch and dermatitis [23,24,25]. However, no studies have determined the effect of BoNT/B1 in pruritus so far.

### 3.3. Duration of Action of BoNT

Activity of BoNT is attributed to neuronal cell entry by the toxin, release of the light chain (LC) into the cells cytosol, and cleavage of terminal SNAREs by the LC, blocking vesicular transmitter release [13]. The duration of action of BoNT depends on the persistence of the catalytically active intracellular LC [44,45]. While in humans the duration of pharmaceutical BoNTs varies from two to six months, depending on the dose, mice usually recover from paralytic effects after local intramuscular injection within three weeks [26,46]. Similarly, the BoNT/A1 and B1 induced reduction in itch behavior in the murine model used in this study lasted for about two weeks, with mice showing gradual reversal that was complete by day 21 (Figure 3). This similarity in duration and gradual reversal indicates a possibly similar mechanism of action of BoNTs in mast cells as in neurons, or an indirect effect of BoNTs in pruritus due to neuronal release inhibition. 

### 3.4. Possible Mechanism of Action of BoNT/A1 and BoNT/B1 in Reducing Induced Itch

Effects of both the pruritogens, 48/80 and CQ are believed to be mediated by the activation of C-fiber terminals in the epidermis. Compelling evidence suggests the role of TRPV1 and TRPA1 ion channels in these subsets of neurons downstream to histamine and CQ [11,12] to mediate calcium induced activation of SNAREs that mobilize synaptic vesicle release, thereby promoting itch sensation. Patients with AD show intense staining of CGRP and substance P-immunoreactive fibers, and uptake of BoNT has previously shown to inhibit the release of these neurotransmitters. Therefore, the reduction in induced itch behavior in mice by BoNT/A1 and B1 could be at least in part due to an effect of the toxins on the C-fibers, rather than a direct effect on mast cells.

Interestingly, we observed that pre-treatment with both BoNT/A1 and BoNT/B1 impaired 48/80 induced mast cell degranulation in cultured murine and human mast cells, indicating that BoNTs may also have a direct effect on mast cells. This is in agreement with previous experiments conducted by Park and colleagues that showed a decrease in mast cell activity seven days following BoNT/A1 treatment in rat skin tissue [47]. While the mast cell release machinery involves SNAREs, which are the target of BoNTs in neurons, the SNARE isoforms considered to be required for mast cell degranulation (SNAP-23 and VAMP-7 and 8) are insensitive to BoNT/A1 and /B1 [28,48,49]. In our study, very low levels of SNAP-25 mRNA expression were observed in both murine and human mast cells, with immunohistochemistry studies confirming the findings for both SNAP-25 and VAMP-1/2/3 and indicating cleavage of these SNARE isoforms by BoNT/A1 and B1. However, western blot data suggested absence or very low levels of SNAP-25 in mast cells, and levels of the BoNT/B1 sensitive VAMP-1/2/3 in mast cells appeared to be unaffected by BoNT/B1. Similar discrepancy in the SNAP-25 expression in mast cells using various detection methods has been previously reported [50]. This result, although confounding, is intriguing and leads to the speculation that BoNTs may utilize a non-canonical mechanism other than SNARE cleavage to inhibit release of secretory granules from mast cells. For example, one possibility could be hindrance in the trafficking of membrane proteins such as TRP receptor subunits to the plasma membrane of mast cells. The role of BoNT/A1 in inhibiting TRPV1 receptor function by affecting regulated endocytosis and reduction in TRPV1 receptor expression has been previously demonstrated in the trigeminal as well as in suburothelial nerve fibers [51,52] (Shimizu et al., Apostolidis et al.). Furthermore, studies have shown that 48/80 degranulation of mast cells employ calcium induced exocytosis in mast cells [53] and BoNTs primarily inhibit the normal depolarization- evoked calcium currents [54]. More studies are required to elucidate the inhibitory mechanism of botulinum toxins on mast cell degranulation and whether the observed in vivo effects are due to direct or indirect action of BoNTs on mast cells.

## 4. Materials and Methods 

### 4.1. Animals

Adult male C57Bl/6 mice, 25–30 g (Harlan Sprague Dawley Inc., Indianapolis, IN, USA), were housed in the vivarium for a minimum of 2 days before use, maintained on a 12/12-h day-night cycle and given free access to food and water. All studies were carried out according to protocols approved by the Institutional Animal Care and Use Committee of the University of California, San Diego, CA, USA. Ethical approval code and date: S00137M and 26 March 2015 (IACUC).

### 4.2. Drugs

Drugs employed were compound 48/80 (48/80) (1 mg/mL) or CQ (2 mg/mL) (Sigma Aldrich, St Louis, MO, USA). BoNT/A1 (Botox©, onabotulinumtoxin A, Allergan Inc., Carlsbad, CA, USA) and BoNT/B1 (Myobloc©, Rimabotulinumtoxin B, Solstice Neurosciences, Louisville, KY, USA) solutions were prepared from stock solutions of 50 U/mL and 5000 U/mL, respectively. These products were then serially diluted to the final concentration in 0.9% saline. All solutions were stored at 4 °C and brought to room temperature prior to use. 

### 4.3. Drug Delivery

Mice were anesthetized (2.5% isoflurane, with 80% oxygen and 20% room air) and were shaven on the dorsolateral aspect of the neck and upper shoulder. Using a 29 G needle (insulin syringe) intradermal injection of 50 µL of BoNT/A1 or BoNT/B1 (1.5 U) or saline was administered. Intradermal injections of 48/80 or CQ (50 µL) were administered on the day of behavioral testing.

### 4.4. Behavior

On the day of testing, animals were placed in a plexiglass cylindrical chamber and a detection band was placed around the hind paw ipsilateral to the shaven area. To initiate scratching behavior, intradermal (ID) injection of 48/80 or CQ was administered in the middle of the shaven area of skin using a 29 G needle. The itch behavior is recorded over the period of 40 min using a paw motion detector (PMD). The PMD detects the movement of a non-ferrous metal band placed around one hind paw of the rodent (band weight = 0.1 g). The testing apparatus consists of cylindrical chambers (mouse: 8.5 cm diameter/22.5 cm tall). Under each cylinder is a pair of circular concentric electromagnetic coils, which serve respectively as antennae for transmission and reception. The transmitter coil assembly emits a 5–8 mW, 6–8 kHz, sinusoidal electromagnetic field. The detection principal is that Eddy currents created by the movements of the ferrous and nonferrous metals perturb the EM field. Such perturbations are detected and produce an output waveform [55,56]. Data were acquired electronically.

### 4.5. Mast Cell Culture

Primary murine MCs were generated from C57BL/6 mouse bone marrow and cultured in RPMI 1640 medium (Life Technologies, Carlsbad, CA, USA) supplemented with 10% heat-inactivated fetal bovine serum (Life Technologies, Carlsbad, CA, USA), 25 mM HEPES (pH 7.4), 4 mM l-glutamine, 0.1 mM nonessential amino acids, 1 mM sodium pyruvate, 50 μM 2-mercaptoethanol, 100 IU/mL penicillin, and 100 μg/mL streptomycin. Recombinant murine IL-3 (1 ng/mL, R&D Systems, Minneapolis, MN, USA) and recombinant murine stem cell factor (20 ng/mL, R&D Systems, Minneapolis, MN, USA) were also included to allow for in vitro differentiation. After 4 weeks, the MCs were fully differentiated, as confirmed by the expression of CD117 (c-Kit) and FcεRI. Cell maturation was confirmed by metachromatic staining with toluidine blue. The purity of MCs was greater than 98%. For the detailed procedure, see [57]. 

Primary human MCs were derived from human cord blood CD34+CD45+ cells from healthy donors (STEMCELL Technologies, Seattle, WA, USA) according to Kirshenbaum and Metcalfe [58]. They were cultured in Stemline II hematopoietic stem cell medium (Sigma Aldrich, St. Louis, MO, USA) with recombinant human SCF and IL-6 (100 ng/mL, Peprotech, Rock Hill, NJ, USA) for 9 weeks. MC differentiation was confirmed by CD117 (c-Kit) and FcεRI expression, and maturation was confirmed by metachromatic staining with toluidine blue. The purity of MCs was greater than 98%.

### 4.6. Mast Cell Degranulation Assay

Degranulation was assessed by measuring the activity of β-hexosaminidase in the supernatants of 1 × 10^5^ MCs in 200 µL Tyrode’s buffer (0.1% BSA, 0.1% glucose, 2 mmol/L MgCl_2_, 137.5 mmol/L NaCl, 12 mmol/L NaHCO_3_, 2.6 mmol/L KCl, pH 7.4) incubated for 24 h with 0.5 U of BoNT-A1 or -B1 before the addition of 1 μg/mL 48/80 (Sigma Aldrich, St Louis, MO, USA). For comparison of mast cell degranulation by 48/80 to CQ, 10 μg/mL 48/80 was used. For each sample assayed, supernatant aliquots (20 μL) were mixed with substrate solution (100 μL) which consisted of 10 mM 4-methylumbelliferyl-2-acetamide-2-deoxy-b-d-glucopyranoside (EMD Millipore, Billerica, MA, USA) in 0.1 M sodium citrate buffer (pH 4.5) and were incubated for 2 h at 37 °C in the dark. The reaction mixtures were excited at 365 nm and measured at 460 nm in a fluorescence plate reader (Gemini EM microplate spectrofluorometer; Molecular Devices, Sunnyvale, CA, USA). To determine the total cellular content of this enzyme, an equivalent number of cells were lysed with 1% Triton X-100 (Sigma Aldrich, St Louis, MO, USA). Release of β-hexosaminidase was calculated as the percentage of the total enzyme content.

### 4.7. Immunohistochemistry on Mast Cells

Mast cells were attached to a glass slide by using Shandon Cytospin 2 cytocentrifuge (Thermo Fisher Scientific, Waltham, MC, USA). The cells were stained with 1 mg/mL anti BoNT/A1-cleaved-SNAP-25 Ab, which recognizes only the BoNT/A1cleavage product of SNAP-25 and not the full-length SNAP-25, and anti-VAMP-2 Ab (Synaptic Systems, Goettingen, Germany) according to the manufacturer’s instructions. Slides were mounted in ProLong Anti-Fade reagent with DAPI (Molecular Probes, Eugene, OR, USA). We imaged the cells using the Bx51 research microscope (Olympus, Center Valley, PA, USA) and X-Cite 120 fluorescence illumination systems (EXFO Photonic Solutions, Mississauga, ON, Canada).

### 4.8. mRNA Isolation and Real-Time Quantitative PCR

Total RNA was isolated using Trizol Reagent (Invitrogen, Carlsbad, CA, USA) and 1 μg of total RNA was used for cDNA synthesis by using iScript cDNA Synthesis Kit (Bio-Rad Laboratories, Hercules, CA, USA) according to the manufacturer’s instructions. cDNA was amplified using Real time-PCR in an ABI 7300 Real-Time PCR system (Applied Biosystems, Foster City, CA, USA). RNA analysis reagents (SYBR Green Master Mix) were from Bio-Rad, Hercules, CA, USA. We used the comparative ∆∆ cycle threshold method to quantify gene expression. Target gene expression levels in the test samples were normalized to the endogenous reference glyceraldehyde-3-phosphate dehydrogenase (GAPDH) (F: 5′-CCA ACC GCG AGA AGA TGA CC-3′ and R: 5′-GAT CTT CAT GAG GTA GTC AGT-3′) levels and reported as the fold difference relative to GAPDH gene expression in untreated baseline control. All assays were performed in triplicate and the experiments were repeated at least three times.

### 4.9. Western Blot Analysis 

Following 24 h treatment with BoNT-A1 or BoNT-B1 on murine and human mast cells, cell lysates were prepared by solubilizing cells in RIPA buffer (Life Technologies, Carlsbad, CA, USA) with protease inhibitor cocktail (Sigma Aldrich, St Louis, MO, USA), at 1 × 10^7^ cells/mL. Cells were incubated for 1 h on ice for complete lysis, and the lysates clarified by centrifugation at 4 °C, for 10 min at 12,000 RPM. Supernatants were collected and stored on ice for immediate use, or at –80 °C until needed. Total protein concentration of the clarified cell lysates was determined by BCA protein assay (Life Technologies, Carlsbad, CA, USA) prior to loading on a gel.

For the Western blot analysis, five micrograms of total cell lysate of each sample were separated on a 12% Bis-Tris NuPAGE gel with MES running buffer (all from Life Technologies). For the mouse spinal cord cell lysate (SC) controls, primary mouse spinal cord cell lysates were prepared as previously described [45], and 8 µl of untreated primary mouse spinal cord cell lysates were used. Proteins were transferred to a PVDF membrane (Millipore 0.45 micron for the SNAP-25 blots, and GE Healthcare (Little Chalfont, UK) 0.2 micron for the VAMP blots) by semi-dry transfer. The membranes were probed with antibodies to beta-actin (Abcam, Cambridge, UK) and VAMP-1/2/3 (Synaptic Systems) (top gel), a polyclonal anti-SNAP-25 antibody (Synaptic Systems) (middle gel), or a monoclonal anti-SNAP-25 antibody (Synaptic Systems) (bottom gel). Images were obtained using PhosphaGlo reagent (KPL, Gaithersburg, MD, USA) and a Fotodyne/FOTO/Analyst FX imaging system (Fotodyne, Hartland, WI, USA).

### 4.10. Statistical Analysis

The data for each variable was put in tabular form (i.e., Excel worksheet). Summary statistics were computed and include group means and standard deviations and numbers of animals per group. Statistical analysis was performed using GraphPad Prism 6, v6.0c (GraphPad Software, San Diego, CA, USA). For comparison of 48/80 and CQ induced scratching, results were compared using a one-way ANOVA across doses or time. Bonferroni post hoc tests were used to compare groups at similar doses or times. For all post hoc comparisons, multiplicity adjusted *p*-values were calculated. In each case, Bonferroni post hoc tests (e.g., *t*-tests with Bonferroni corrections) were undertaken and presented in the graphics and figure legends for values between *p* < 0.01 and *p* < 0.0001.
